# Monte Carlo based dosimetry of extraoral photobiomodulation for prevention of oral mucositis

**DOI:** 10.1038/s41598-023-47529-3

**Published:** 2023-11-22

**Authors:** Anna N. Yaroslavsky, Tyler W. Iorizzo, Amy F. Juliano, Ather Adnan, James D. Carroll, Stephen T. Sonis, Christine N. Duncan, Wendy B. London, Nathaniel S. Treister

**Affiliations:** 1 Advanced Biophotonics Laboratory, Department of Physics and Applied Physics, University of Massachusetts Lowell, Lowell, MA 01854 USA; 2 Department of Dermatology, Massachusetts General Hospital, Boston, MA 02114 USA; 3 IPG Medical, Marlborough, MA 01752 USA; 4 Department of Radiology, Massachusetts Eye and Ear, Harvard Medical School, Boston, MA 02114 USA; 5 College of Medicine, Texas A&M Health Science Center, Houston, TX 77030 USA; 6 THOR Photomedicine, Amersham, HP7 9FB UK; 7 Department of Surgery, Division of Oral Medicine and Dentistry, Brigham and Women’s Hospital, Boston, MA 02114 USA; 8 Department of Oral Medicine, Infection and Immunity, Harvard School of Dental Medicine, Boston, MA 02114 USA; 9 Biomodels LLC., Waltham, MA 02451 USA; 10 Department of Pediatrics, Dana-Farber/Boston Children’s Cancer and Blood Disorders Center, Harvard Medical School, Boston, MA 02114 USA

**Keywords:** Computational biophysics, Optics and photonics, Translational research

## Abstract

Photobiomodulation therapy (PBMT) is recommended for prevention and treatment of oral mucositis, a painful condition that occurs in cancer patients. Intraoral PBMT is limited to treating distal oral mucosa and oropharynx. Extraoral PBMT may provide a more efficient intervention. The goal of this study was to develop a clinically viable protocol for extraoral PBMT. Monte Carlo modeling was used to predict the distribution of 850 nm light for four treatment sites, using anatomical data obtained from MRI and optical properties from the literature. Simulated incident light power density was limited to 399 mW/cm^2^ to ensure treatment safety and to prevent tissue temperature increase. The results reveal that total tissue thickness determines fluence rate at the oral mucosa, whereas the thickness of individual tissue layers and melanin content are of minor importance. Due to anatomical differences, the fluence rate varied greatly among patients. Despite these variations, a universal protocol was established using a median treatment time methodology. The determined median treatment times required to deliver efficacious dose between 1 and 6 J/cm^2^ were within 15 min. The developed PBMT protocol can be further refined using the combination of pretreatment imaging and the Monte Carlo simulation approach implemented in this study.

## Introduction

Photobiomodulation therapy (PBMT) is an optical treatment that utilizes red—near infrared (NIR) light to produce anti-inflammatory and analgesic effects for various medical conditions^1,2^. The mechanisms of PBMT occur within the mitochondria of cells, where treatment light is absorbed by cytochrome C oxidase^1^. This leads to a modulation of oxidative stress through nitric oxide release, which increases adenosine triphosphate (ATP) production, regulation of reactive oxygen species, and subsequent regulation of NF-kB^3^. Immune cell activity is enhanced after light exposure, further contributing to the analgesic effects of PBMT^4,5^. PBMT stimulates all cell types, both cancerous and healthy^6–11^. Therefore, it is important to complete all medical procedures and confirm the patient is cancer free prior to enrolling in PBMT.

Oral mucositis (OM) is a common toxicity among patients undergoing myeloablative hematopoietic cell transplantation (mHCT)^12^. Clinical features include painful oral ulcerations which can lead to the inability to eat, drink, or swallow^13^. Management of OM may require opioid analgesia and parenteral nutrition, and the length of hospital stay as well as overall costs may increase^14^.

PBMT protocols and guidelines have been established for OM prevention and management by several national and international professional organizations^2,12,15–17^. Light is usually delivered intraorally using either 632.8 nm He–Ne gas lasers or 660 nm diode lasers in a spot-by-spot manner directly to the mucosa tissue^2^. These procedures can be lengthy, uncomfortable, and logistically challenging^18^. Treatment can be difficult to deliver when using intraoral PBMT. Intraoral delivery also limits treatment to the distal oral mucosa and oropharynx, which are frequently affected by OM. Extraoral, or transcutaneous PBMT may provide treatment in a simpler, more comfortable, and more effective application and would provide a broader treatment area to allow inclusion of the oral cavity, the oropharynx, and the upper esophagus. This would provide a particularly ideal approach for pediatric patients where cooperation and tolerability of intraoral treatment may be challenging.

Universal treatment protocols have not been established for extraoral PBMT^19^. Studies to date investigating extraoral PBMT for OM prevention provide no rationale for the parameters used. Extraoral applications need to account for the attenuation of light through the tissue layers of the target site. To increase penetration depth, extraoral protocols would require longer wavelengths compared to intraoral procedures (i.e., 800–900 nm). Extraoral PBMT also requires incident power densities higher than the 24–31.25 mW/cm^2^ sometimes used for intraoral PBMT to ensure delivery of an equivalent efficacious dose^20,21^. However, the incident power density needs to be within the safety limits defined by American National Standards Institute (ANSI)^22^ to avoid excessive heating of the target area.

The objective of this study was to develop an evidence-based extraoral PBMT treatment protocol for prevention of OM. Morphological data was used to model multiple treatment sites, while Monte Carlo simulations predicted the light distribution from extraoral PBMT application. We previously demonstrated that Monte Carlo simulations were within 12% accuracy compared to in vivo transmittance measurements^23^. Simulation results were used to determine the treatment parameters such that the efficacious dose is safely delivered to the oral mucosal tissue in a reasonable treatment time.

## Results

Propagation of 850 nm light was simulated through several treatment sites, including the cheek, lip (anterior midline), mandible angle, and neck (anterior midline). Anatomical data from archival MRI scans of 18 study subjects was used as a model. The incident power density was set to 399 mW/cm^2^, which corresponds to the maximal incident power density for 850 nm light allowed by ANSI standards for skin^22^. A summary of the simulation results for subjects who had the minimum and maximum treatment site thicknesses is presented in Table 1. Detailed information for each study subject is provided in Supplementary Tables S1–S4.

**Table 1:** Results summary.

Treatment site	Subject #/age/gender	Tissue thickness, mm				Total thickness, mm	Fluence rate at buccal surface, mW/cm^2^	Average absorbed power, mW/cm^3^
		Skin µ_a_ = 0.013/mm, µ_s_' = 1.85/mm^34^	Fat µ_a_ = 0.009/mm, µ_s_' = 1.1/mm^34^	Muscle µ_a_ = 0.035/mm, µ_s_' = 0.65/mm^34^	Cartilage µ_a_ = 0.018/mm, µ_s_' = 0.36/mm^35^			
Cheek	#10/11/F	1	8	4	0	13	10.0	2.0
	#13/16/F	1	20	8	0	29	0.4	0.9
Lip (anterior midline)	#6/7/F	1	2	3	0	6	36.3	3.7
	#7/10/M	2	1	3	0	6	30.3	4.2
	#16/17/M	2	5	7	0	14	5.6	2.1
Mandible angle	#15/16/F	1	2	3	0	6	35.5	1.5
	#18/20/F	1	15	5	0	21	2.4	0.6
Neck (anterior midline)	#1/5/F	1	2	2	4	9	26.3	1.9
	#14/16/F	1	6	9	5	21	2.0	0.9
	#18/20/F	2	7	4	8	21	3.2	0.8

Patient demographic, anatomical data, and simulation results for study subjects with minimum and maximum treatment site thicknesses. Optical properties were obtained from the literature^34,35^. Anisotropy factors for all tissues were set to 0.9.

### Anatomical data from archival MRI studies

**Figure 1:**
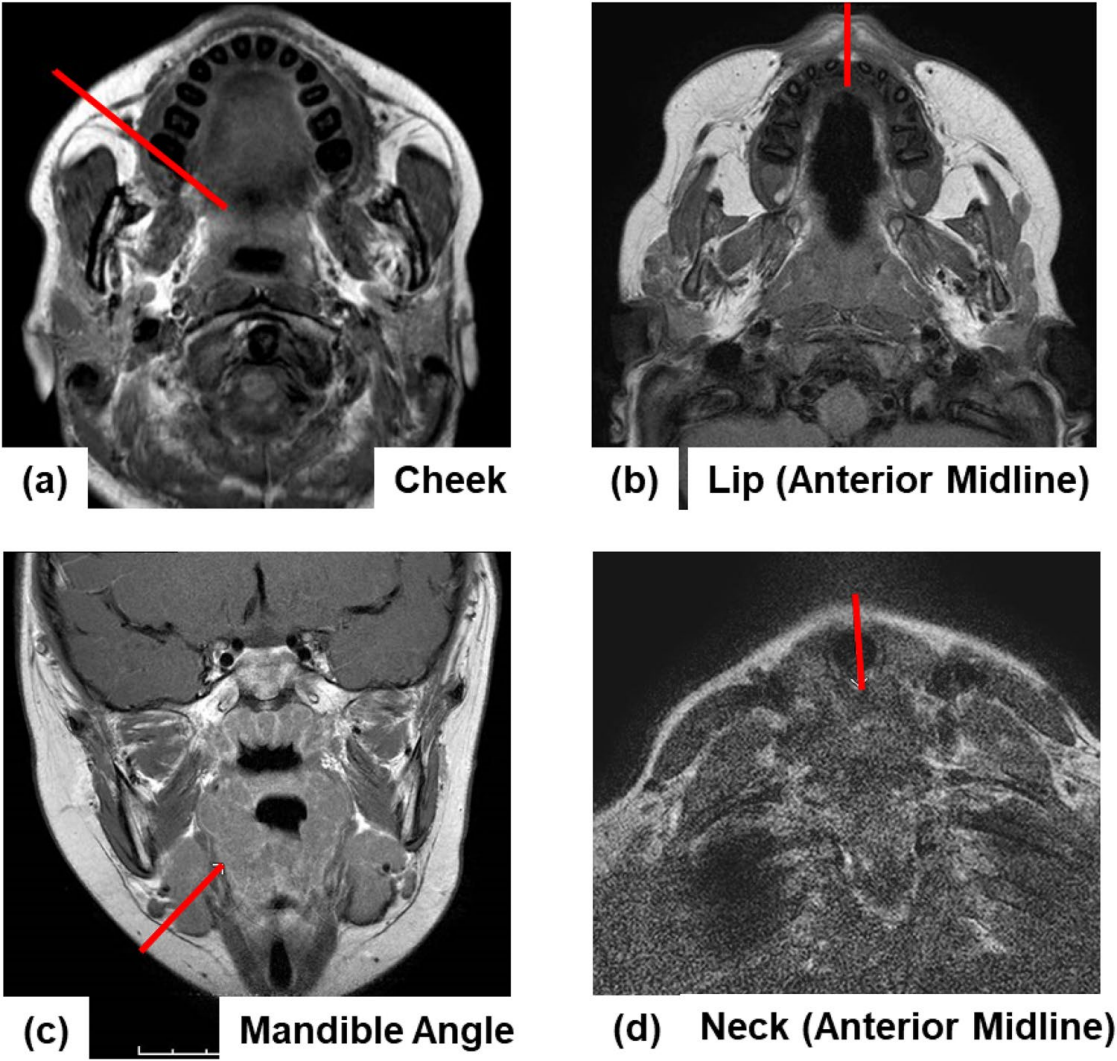
Example MR Images. Example MR images of cheek (**a**), lip (anterior midline) (**b**), mandible angle (**c**), and neck (anterior midline) (**d**) treatment sites. Tissue types and thicknesses were recorded along each trajectory indicated by the red line.

For each treatment site, the distance between the point of light entry into the skin and its exit at the inner aerodigestive tract mucosal surface along a straight line was determined from MR images using anatomical landmarks for standardization purposes. Example MR images are presented in Fig. 1. For the cheek treatment site (Fig. 1a), the axial image that contained Stensen’s duct was selected for the measurements. A line perpendicular to the tangent to the cheek surface intersecting the oral opening of Stensen’s duct was drawn, and the thickness of each soft tissue layer along this line was obtained. For the lip (anterior midline) treatment site (Fig. 1b), that same image that contained Stensen’s duct was used for measurements, which approximated a level just superior to the upper lip in the region of the philtrum. A line along the sagittal plane of the patient in the anterior midline was drawn, and the thickness of each soft tissue layer along this line was obtained. For the mandible angle treatment site (Fig. 1c), the coronal image that displayed the bulk of the submandibular gland was selected. A line perpendicular to the tangent of the skin surface at the level of the angle of the mandible coursing toward the mucosal surface of the lateral oral tongue/floor of mouth mucosa was drawn, and the thickness of each soft tissue layer along this line was obtained. For the neck (anterior midline) treatment site (Fig. 1d), the axial image at the level of the cricoid ring was selected. A line along the sagittal plane of the patient in the anterior midline was drawn, and the thickness of each soft tissue layer along this line was obtained. Tissue layers considered for each treatment site are presented in Table 1. Measurements for each subject are presented in Supplementary Tables S1–S4. Total tissue thickness ranged from 13 to 29 mm for the cheek, 6–14 mm for the lip, 6–21 mm for the mandible angle, and 9–21 mm for the neck.

### Modeling results

**Figure 2:**
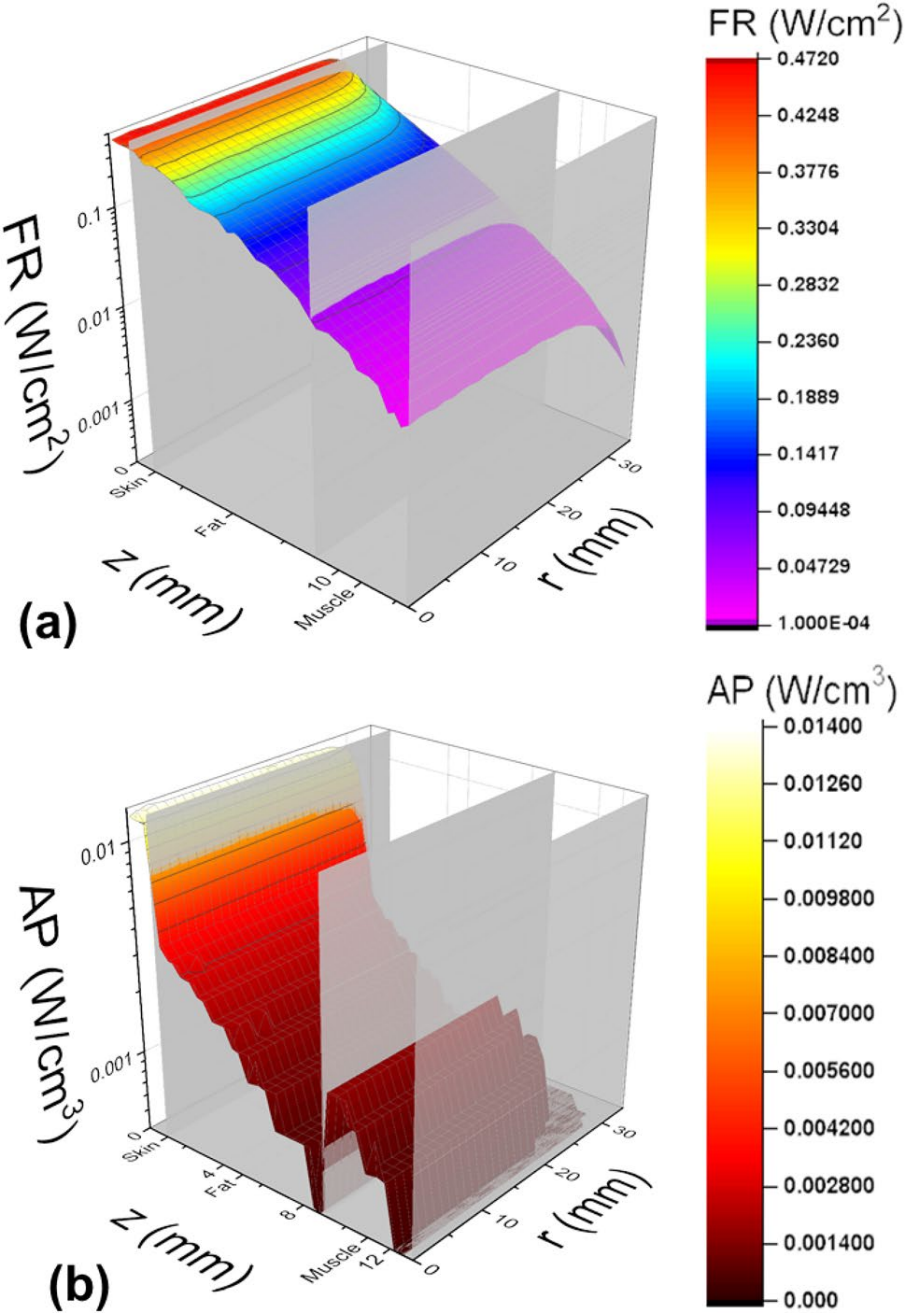
Fluence Rate and Absorbed Power for Cheek. Fluence rate (**a**) and absorbed power (**b**) distributions for Subject #10 (thinnest cheek). The vertical axis corresponds to fluence (**a**) or absorbed power (**b**). The z-axis shows treatment volume depth, and the r-axis is the radial distance from the center of the treatment beam.

**Figure 3:**
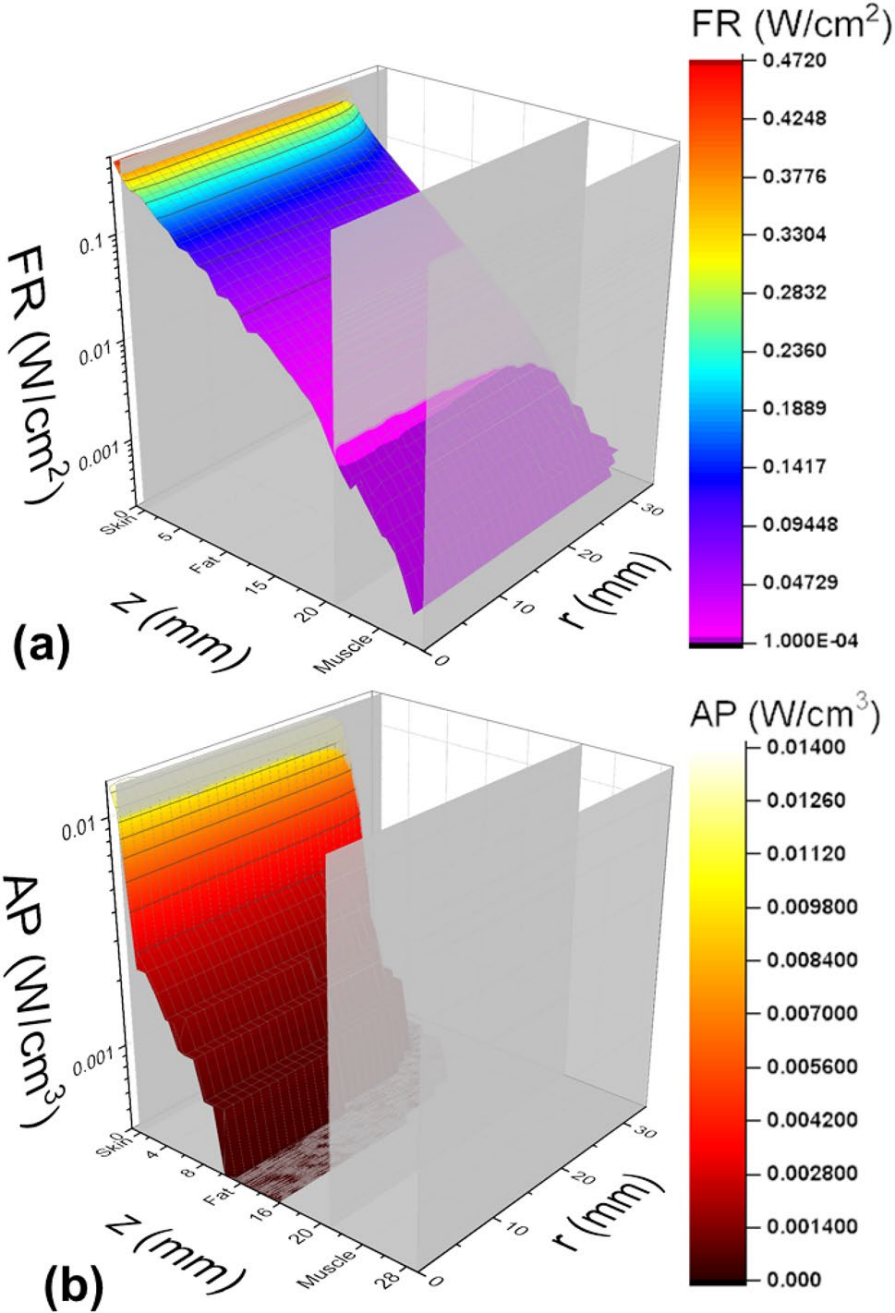
Fluence Rate and Absorbed Power for Cheek. Fluence rate (**a**) and absorbed power (**b**) distributions for Subject #13 (thickest cheek). The vertical axis corresponds to fluence (**a**) or absorbed power (**b**). The z-axis shows treatment volume depth, and the r-axis is the radial distance from the center of the treatment beam.

Subject #10, 11-year-old female, had the thinnest cheek, whereas Subject #13, 16-year-old female, had the thickest cheek (Table 1, rows 1 and 2). For both subjects, skin was 1 mm thick (column 3). The fat tissue layer (column 4) was 8 and 20 mm, while the muscle layer (column 5) was 4 and 8 mm for the thinnest and thickest cheeks, respectively. The fat tissue layer was found to have the largest variation among the subjects (range = 12 mm), followed by muscle (range = 4 mm). This resulted in the total cheek thickness (Table 1, column 7) to vary between 13 and 29 mm (range = 16 mm). Simulated fluence rates, defined as the total radiant power density incident on a cross-sectional area^24^, are shown in Figs. 2a and 3a. The lowest, 0.4 mW/cm^2^, and the highest, 10.0 mW/cm^2^, fluences rates at the buccal surface corresponded to Subject #13 with the thickest cheek and to Subject #10 with the thinnest cheek, respectively. The fluence rates varied by a factor of 25.

Averaged absorbed powers (Figs. 2b and 3b) for Subjects #10 and #13 are presented in Table 1 (column 9, rows 1 and 2). Values were calculated by averaging the total absorbed power over the entire treatment volume. Absorbed power ranged between 0.9 and 2.0 mW/cm^3^, varying by a factor of 2.2. Subjects with thinner treatment sites experienced higher absorbed power levels compared to those with thicker treatment sites. Predictably, light was attenuated less within thinner treatment sites. This allowed higher fluence rates to reach deeper tissues. Due to low absorption, there was no observable temperature increase in any tissue including the skin’s surface.

All treatment sites (Table 1, rows 3–10) exhibited similar dependencies of fluence rate and absorbed power distribution to those determined for the cheek (Supplementary Figs. S1–S6). The cheek treatment site exhibited the largest variations (range of 16 mm), whereas the lip site exhibited the smallest differences in overall tissue thickness (range of 8 mm). Skin, while the most attenuating tissue of those presented in Table 1, had a thickness between 1 and 2 mm in all subjects. Thus, differences of the fluence rate at the buccal surface were majorly impacted by variation in fat and muscle thicknesses. It can be readily appreciated that the large variation in overall tissue thickness within each treatment site and across the sites yields orders of magnitude variations in fluence rates.

To investigate the impact of different tissue types on the resulting fluence at the buccal mucosa, we calculated and analyzed absolute and relative transmittance through each tissue layer for the subjects with the same overall lip treatment site thickness. Fluence rates at the boundary of each tissue layer are shown in Table 2. Relative fluence quantifies percent transmission through each individual layer, whereas absolute fluence provides total transmission. Subjects #6 and #7 had the same lip thickness of 6 mm. Skin thickness of Subjects #6 and #7 was 1 mm and 2 mm, respectively. As a result, fluences at the skin-fat boundaries for Subjects #6 and #7 were 97.7% and 55.6%, respectively. However, even though the scattering coefficient of skin was almost two times higher than that of fat and three times higher than that of muscle, transmittance through all the tissue layers, including skin, fat, and muscle yielded similar fluence rates of 9.7% and 8.2%, respectively. This can be explained by relatively small overall thickness of skin tissue layer (1–2 mm), as compared to combined fat and muscle tissues (4–5 mm).

**Table 2:** Relative and absolute transmittance.

Fluence rate transmittance						
Lip site						
Tissue	Subject #6, 7 years old, female			Subject #7, 10 years old, male		
	Thickness (mm)	Relative	Absolute	Thickness (mm)	Relative	Absolute
Skin	1	97.70%	97.70%	2	55.60%	55.60%
Fat	2	42.80%	41.80%	1	63.80%	35.50%
Muscle	3	23.20%	9.70%	3	23.00%	8.20%

Relative and absolute transmittance through the lip site.

### Impact of melanin

To analyze the impact of different melanin content on the fluence rate, simulations were performed for skin types I (fair) and VI (dark)^25,26^. Fluence rates for subjects with fair and dark skin with maximum and minimum total tissue thickness are presented and compared in Table 3. Optical properties shown in Table 3 (row 1, columns 3 and 4) indicate that due to presence of melanin, absorption in fair skin is 3.8 times lower as compared to that of dark skin.

**Table 3:** Melanin impact.

Treatment site	Subject#/age/gender	Fluence rate at buccal surface, mW/cm^2^		
		Type I skin µ_a_ = 0.013/mm, µ_s_' = 1.85/mm^34^	Type VI skin µ_a_ = 0.050/mm, µ_s_' = 2.00/mm^34^	% Difference
Cheek	#10/11/F	10	8.2	19.8%
	#13/16/F	0.4	0.4	0.0%
Lip	#6/7/F	36.3	30.1	18.7%
	#16/17/M	5.6	3.9	35.8%
Mandible angle	#15/16/F	35.5	29.7	17.8%
	#18/20/F	2.4	1.9	23.3%
Neck	#1/5/F	26.3	24.3	7.9%
	#18/20/F	3.2	2.4	28.6%

Fluence rates at the buccal surface of subjects with maximum and minimum total tissue thicknesses for type I skin and type VI skin.

Subjects with 2 mm of skin (Subject #16, lip, and Subject #18, neck) presented a larger decrease in fluence rate for skin type VI as compared to skin type I. Specifically, fluence rate decreased by 35.8% and 28.6% for Subjects #16 and #18, respectively. In contrast, for the subjects with skin thickness of 1 mm, fluence rate decreased by at most 23.3% (Subject #18, mandible angle). Notably, the cheek site of Subject #13 did not present any decrease in fluence rate due to presence of melanin. This may be explained by the negligibly small thickness of skin layer (1 mm) as compared to overall thickness of the cheek (29 mm). This result confirms that even though skin is the most attenuating tissue layer, fluence attenuation is primarily determined by the overall thickness of all tissues along the light trajectory.

### Median dose parameters

Personalized treatment planning is one method that would account for differences in patient anatomy. However, this would require imaging the patient to identify and measure tissue thicknesses, and to perform Monte Carlo simulations to predict fluence rate distribution and treatment times. To establish a universal treatment protocol, a median dose approach was used (Table 4). Table 4 column 2 shows the median doses delivered within 1 min of 399 mW/cm^2^ irradiation. The cheek (row 2), which had some of the largest site thicknesses, had a median dose less than 0.5 J/cm^2^, whereas the lip, having some of the smallest site thicknesses, had a median dose greater than 2 J/cm^2^. The median treatment times for each treatment site, assuming an efficacious dose of 2 J/cm^2^^20,27^, are presented in column 3. Treatment times for all sites were within 15 min. The cheek had the highest treatment time of 11 min. Treatment times were less than 2 min for all other sites and less than 1 min for the lip.

**Table 4:** Median treatment times.

Site	Median dose in 1 min (J/cm^2^)	Median treatment time (min)
Cheek	0.18 (0.16–0.308)	11.1 (6.49–12.5)
Lip	2.4 (1.94–3.14)	0.833 (0.637–1.03)
Mandible angle	1.68 (1.2–2.36)	1.19 (0.847–1.67)
Neck	1.2 (0.84–2.02)	1.67 (0.990–2.38)

Median dose and treatment time for each treatment site. Interquartile ranges are shown in parentheses. An efficacious dose of 2 J/cm^2^ was assumed when calculating treatment times. Median dose and treatment times presented account for all skin types.

## Discussion

This study reports on the development of an extraoral PBMT treatment protocol for preventing and/or treating oral mucositis. To the best of our knowledge, this is the first study that employed Monte Carlo modeling, archival MRI scans, and optical properties data to calculate fluence rate and absorbed power distributions to determine the parameters required to achieve a safe and efficacious dose to the oral mucosa for several prospective treatment sites.

Treatment times were established for the cheek, lip, mandible angle, and neck sites. The side of the neck was also considered for extraoral PBMT. However, large blood vessels significantly attenuated light, preventing the treatment light from reaching the target mucosa.

Common tissue layers among the four treatment sites were skin, fat, and muscle (Table 1). Of these, skin has the highest attenuation properties. However, due to its smallest thickness of 1–2 mm, its impact on the variation in the fluence rate at the mucosa was less than 40%. In contrast, fat has the lowest attenuation, but exhibited the greatest variation in thickness between 1 and 20 mm (Table 1). As attenuation increases with tissue thickness, fluence rates were strongly affected by these variations and were on the order of 760% (Table 1). It can also be appreciated that subjects with similar total site thicknesses exhibited similar fluence rates, regardless of differences in individual tissue layers (Table 2). Thus, the total thickness of the treatment site was found to have the greatest impact on the delivered dose to mucosal tissue.

Fluence rates differed by at most 23.3% and 35.8% for subjects with 1 mm and 2 mm skin layer, respectively when melanin concentrations varied between types I and VI. Thus, impact of melanin presence was minor as compared to that of the total tissue thickness. It should also be noted that for the simulations it was assumed that melanin was evenly distributed through the skin, whereas melanocytes are located at depths of 35–40 µm within the skin^28^. Thus, differences in fluence rate due to melanin absorption and scattering may be even smaller than those estimated here.

The performed Monte Carlo simulations also monitored the resulting absorbed power distribution within each patient across all treatment sites (Table 1). Absorbed power levels were highest at the skin’s surface, and generally decreased with tissue depth. A local maximum occurred at fat–muscle boundaries. This can be explained by a significant increase in absorption coefficient of muscle as compared to fat. For the simulations we have used the maximum power density for skin defined by the ANSI Standards. Therefore, low total absorbed power (Table 1) was observed across all patients. Thus, no temperature changes were recorded.

One of the most important outcomes of this study was that large anatomical variations among the subjects caused orders of magnitude differences in the dose delivered to the oral mucosa. The most accurate method to take these differences into account would be the development of personalized treatment protocols. To implement this approach, an imaging procedure would be required prior to treatment. While MRI was used in this study, other more cost-effective modalities, such as ultrasound, could be employed in practice. The maximum tissue thickness in this study was 2.9 cm. For imaging at such shallow depths high-frequency ultrasound can be utilized to improve spatial resolution from ~ 1 mm down to 0.1 mm. After imaging, Monte Carlo simulations would be performed using the acquired images and optical properties from the literature. As the accuracy of Monte Carlo simulations for this use was previously determined to be at least 88%^23^, the appropriate personalized treatment times for each patient and/or site could be reliably determined from the simulation results.

Clinical implementation of personalized dosimetry will inevitably lead to higher requirements of the skills and training of the medical personnel, as well as to an increase in healthcare costs. Therefore, one of the overarching goals of the study was to propose a viable universal treatment protocol. As the efficacious dose for oral mucositis has been reported to be in the range of 1.0–6.0 J/cm^2^, we proposed the use of and calculated standardized median treatment times (Table 4). These median values will be used as a starting point to develop and refine a treatment protocol in future clinical studies^29–33^.

In conclusion, we have proposed and implemented an evidence-based comprehensive approach to extraoral PBMT dosimetry for the prevention of oral mucositis, which utilized archival MRI data, optical properties of tissues from the literature, and Monte Carlo simulations. We have established universal treatment parameters for extraoral PBMT by calculating median dose to determine the treatment time needed to deliver a dose within efficacious range to each site investigated. With some empirical optimization, the established parameters should provide effective treatment, while not delivering the same dose per patient. More generally, the method conceived and implemented in this study can be applied for the development of personalized treatment protocols for each patient.

## Materials and methods

All methods were performed in accordance with the relevant guidelines and regulations.

### Study design

The objective of the study was to model the dosimetry of extra orally delivered photobiomodulation therapy (PBMT) for the prevention of oral mucositis. This study was approved by Institutional Review Boards of the Dana-Farber/Harvard Cancer Center and Massachusetts Eye and Ear Institute. Archival deidentified magnetic resonance imaging (MRI) scans of the head and neck region from 18 pediatric, adolescent, and young adult patients were used to determine tissue thicknesses from the skin to the mucosal surfaces of the oral cavity, oropharynx, and esophagus. This information was utilized to assign subjects’ anatomical and optical properties for the Monte Carlo technique. Simulations were performed to estimate the dose provided by light energy reaching the target tissues, assuming direct extraoral application. Simulation results were used to determine the treatment time needed to deliver an efficacious dose of 2 J/cm^2^ to mucosa tissue without exceeding 399 mW/cm^2^, which corresponds to the maximal incident power density allowed by ANSI standards for skin^22^.

### Study subjects

MRI scans from eight male and ten female patients, ages ranging between 5 and 20 years old, were used for the study (Table 5). Anatomical details for each study subject are summarized in Supplementary Tables S1–S4.

**Table 5:** Patient demographics.

Characteristic	Mean (range) (N = 18)
Age of patient, years	11.8 (5–20)
**Sex of patient**	
Male, number (%)	8 (44)
Female, number (%)	10 (56)
**Cheek treatment site**	
Skin thickness, mm	1.3 (1–2)
Fat thickness, mm	16.3 (6–22)
Muscle thickness, mm	4.8 (2–8)
**Lip (anterior midline) treatment site**	
Skin thickness, mm	1.7 (1–2)
Fat thickness, mm	3.3 (1–7)
Muscle thickness, mm	3.7 (1–7)
**Mandible angle treatment site**	
Skin thickness, mm	1.1 (1–2)
First fat layer thickness, mm	4.3 (1–11)
First muscle layer thickness, mm	1.2 (1–2)
Second fat layer thickness, mm	1.6 (1–6)
Second muscle layer thickness, mm	3.2 (1–6)
**Neck (anterior midline) treatment site**	
Skin thickness, mm	1.1 (1–2)
Fat thickness, mm	2.8 (1–8)
First muscle layer thickness, mm	2.7 (1–6)
Cartilage thickness, mm	5.5 (3–9)
Second muscle layer thickness, mm	1.9 (1–6)

Patient characteristics, anatomy, and tissue thicknesses.

### Archival MR image analysis

Archival MRI studies were acquired using a 3-Tesla MRI axial T1-weighted sequence (3 T Achieva, Philips Healthcare, Best, The Netherlands). Four potential treatment sites were considered: cheek, lip, mandible angle, and neck (Fig. 1). Simulated trajectories were drawn perpendicular to the tangent at the skin’s surface, extending to the oral buccal surface. Tissue types were recorded, and thicknesses were measured using electronic calipers on a standardized PACS viewing station (Synapse, Fuji, Japan). For standardization purposes, uniform anatomic landmarks were used to select the index MR image and to draw the line that best simulated the trajectory of light delivery.

### Optical properties

Optical properties of skin, fat, muscle, cartilage, and blood at 850 nm were obtained from the literature^34–36^. Respective absorption, reduced scattering, and anisotropy factors used are summarized in Table 1 (row 1). To analyze the effects of different melanin concentrations on treatment efficacy, Monte Carlo simulations were performed for skin types I and VI.

### Monte Carlo simulations

A Monte Carlo technique^23,37^ validated in our previous study^23^ was used to simulate the propagation of 850 nm light through each treatment trajectory to calculate the fluence rate.

A parallel plane, multilayered tissue geometry was utilized. Each layer (e.g., skin, fat, muscle, etc.) was divided into voxels and was assigned the absorption, scattering, anisotropy, refractive index, and thickness of their respective tissue type.

The illumination was modeled as a collimated uniform beam with a diameter equal to that of the treatment light source. Photons were assigned an initial weighting factor at the beginning of the simulation. Simulations began by calculating the photon’s path length and scattering angle. Spatial and angular distributions of the simulated light were assumed to be radially symmetric. As the photon approached a tissue boundary, the Monte Carlo technique calculated whether transmittance would occur. Fresnel formulas were used to calculate the probability of the photon reflecting. If the photon crosses the boundary, Snell’s law was used to determine the new direction and path length. A portion of the photon’s weight was then subtracted and quantified as energy deposited in the voxel. This process continues until the photon reaches a critical weight. Then, a “Russian Roulette” is played^38^ to determine whether the photon annihilates. After simulating the propagation of several photons, fluence rates were calculated from the logged spatial distributions and absorbed energies.

### Statistical analysis

The delivered dose provided by therapeutic light was determined using Monte Carlo simulations for each application site for skin types I and VI. Median dose, median treatment time, and interquartile ranges were then calculated from the simulation results.

### Institutional review board

This study was approved by the Institutional Review Boards of the Dana-Farber/Harvard Cancer Centre and Massachusetts Eye and Ear (protocol # 16–164, 19 April 2016).

### Informed consent

In accordance with the IRB protocol, written informed consent was obtained from all subjects involved in the study.

### Supplementary Information

Supplementary Information.

## Data Availability

Datasets related to this article can be obtained from the corresponding author.
