# Supplementary material for: Monte Carlo based dosimetry of extraoral photobiomodulation for prevention of oral mucositis
Source: Sci Rep. 2023 Nov 22;13:20425. doi: 10.1038/s41598-023-47529-3 (PMC10665335; doi:10.1038/s41598-023-47529-3)
Supplement: Supplementary file 1 — Supplementary Information. [file 41598_2023_47529_MOESM1_ESM.docx]

Monte Carlo Based Dosimetry of Extraoral Photobiomodulation for Prevention of Oral Mucositis

Anna N. Yaroslavsky, Tyler W. Iorizzo, Amy F. Juliano, Ather Adnan, James D. Carroll, Stephen T. Sonis, Christine N. Duncan, Wendy B. London and Nathaniel S. Treister

**SUPPLEMENTAL INFORMATION**

| Subject # | Age / Gender | Skin, mm | Fat, mm | Muscle, mm | Total Tissue Thickness, mm | Fluence Rate, mW/cm2 | Dose @ 1 min, J |
| --- | --- | --- | --- | --- | --- | --- | --- |
|  |  |  |  |  |  |  |  |
| 1 | 5F | 1 | 22 | 2 | 25 | 1.48 | 0.176 |
| 2 | 6M | 1 | 20 | 4 | 25 | 1.32 | 0.160 |
| 3 | 6F | 1 | 21 | 3 | 25 | 1.40 | 0.168 |
| 4 | 7M | 1 | 13 | 6 | 20 | 2.63 | 0.320 |
| 5 | 7M | 1 | 22 | 2 | 25 | 1.48 | 0.176 |
| 6 | 7F | 1 | 18 | 3 | 22 | 2.27 | 0.272 |
| 7 | 10M | 1 | 21 | 6 | 28 | 0.72 | 0.088 |
| 8 | 10F | 1 | 21 | 5 | 27 | 0.80 | 0.096 |
| 9 | 11M | 1 | 17 | 6 | 24 | 1.32 | 0.160 |
| 10 | 11F | 1 | 8 | 4 | 13 | 9.98 | 1.216 |
| 11 | 14M | 2 | 11 | 4 | 17 | 4.39 | 0.512 |
| 12 | 15F | 2 | 11 | 5 | 18 | 3.39 | 0.408 |
| 13 | 16F | 1 | 20 | 8 | 29 | 0.40 | 0.048 |
| 14 | 16F | 2 | 17 | 3 | 22 | 2.03 | 0.248 |
| 15 | 16F | 1 | 17 | 5 | 23 | 1.80 | 0.216 |
| 16 | 17M | 2 | 13 | 7 | 22 | 1.52 | 0.184 |
| 17 | 19M | 2 | 6 | 8 | 16 | 3.79 | 0.456 |
| 18 | 20F | 2 | 16 | 6 | 24 | 1.16 | 0.136 |

Tissue types and thicknesses were determined from archival MR studies of 18 pediatric subjects and used for Monte Carlo modeling to determine fluence rates within each of the four treatment sites during extraoral PBMT. Supplementary Tables S1 - S4 summarize anatomical information and simulation results for all study subjects and for all treatment sites.

**Table S1.** **Cheek treatment site. Patient demographic, anatomical data and simulation results.**

**Table S2.** **Lip treatment site. Patient demographic, anatomical data and simulation results.**

| Subject # | Age / Gender | Skin, mm | Fat, mm | Muscle, mm | Total Tissue Thickness, mm | Fluence Rate, mW/cm2 | Dose @ 1 min, J |
| --- | --- | --- | --- | --- | --- | --- | --- |
|  |  |  |  |  |  |  |  |
| 1 | 5F | 1 | 5 | 2 | 8 | 26.33 | 3.200 |
| 2 | 6M | 2 | 1 | 4 | 7 | 24.34 | 2.960 |
| 3 | 6F | 2 | 4 | 3 | 9 | 17.56 | 2.080 |
| 4 | 7M | 1 | 4 | 4 | 9 | 19.55 | 2.320 |
| 5 | 7M | 2 | 2 | 5 | 9 | 15.96 | 1.920 |
| 6 | 7F | 1 | 2 | 3 | 6 | 36.31 | 4.400 |
| 7 | 10M | 2 | 1 | 3 | 6 | 30.32 | 3.680 |
| 8 | 10F | 2 | 1 | 4 | 7 | 24.34 | 2.960 |
| 9 | 11M | 2 | 1 | 4 | 7 | 24.34 | 2.960 |
| 10 | 11F | 1 | 2 | 4 | 7 | 28.73 | 3.440 |
| 11 | 14M | 1 | 6 | 4 | 11 | 14.36 | 1.760 |
| 12 | 15F | 1 | 5 | 1 | 7 | 31.52 | 3.760 |
| 13 | 16F | 2 | 3 | 4 | 9 | 16.76 | 2.000 |
| 14 | 16F | 2 | 2 | 4 | 8 | 20.35 | 2.480 |
| 15 | 16F | 2 | 4 | 3 | 9 | 17.56 | 2.080 |
| 16 | 17M | 2 | 5 | 7 | 14 | 5.59 | 0.640 |
| 17 | 19M | 2 | 7 | 4 | 13 | 8.38 | 1.040 |
| 18 | 20F | 2 | 5 | 3 | 10 | 14.36 | 1.760 |

**Table S3.** **Mandible angle treatment site. Patient demographic, anatomical data and simulation results.**

| Subject # | Age / Gender | Skin, mm | Fat, mm | Muscle, mm | Fat, mm | Muscle, mm | Total Tissue Thickness, mm | Fluence Rate, mW/cm2 | Dose @ 1 min, J |
| --- | --- | --- | --- | --- | --- | --- | --- | --- | --- |
|  |  |  |  |  |  |  |  |  |  |
| 1 | 5F | 1 | 7 | 1 | 1 | 1 | 11 | 15.16 | 1.840 |
| 2 | 6M | 1 | 1 | 1 | 1 | 4 | 8 | 23.14 | 2.800 |
| 3 | 6F | 1 | 7 | 1 | 1 | 1 | 11 | 15.16 | 1.840 |
| 4 | 7M | 1 | 3 | 1 | 1 | 6 | 12 | 9.18 | 1.120 |
| 5 | 7M | 1 | 5 | 1 | 1 | 2 | 10 | 16.76 | 2.000 |
| 6 | 7F | 1 | 6 | 1 | 6 | 5 | 19 | 2.79 | 0.320 |
| 7 | 10M | 1 | 4 | 1 | 1 | 4 | 11 | 13.17 | 1.600 |
| 8 | 10F | 1 | 5 | 1 | 1 | 3 | 11 | 13.17 | 1.600 |
| 9 | 11M | 1 | 1 | 2 | 1 | 3 | 8 | 22.34 | 2.720 |
| 10 | 11F | 1 | 6 | 1 | 1 | 3 | 12 | 11.97 | 1.440 |
| 11 | 14M | 1 | 1 | 1 | 2 | 5 | 10 | 13.97 | 1.680 |
| 12 | 15F | 1 | 5 | 1 | 2 | 2 | 11 | 13.97 | 1.680 |
| 13 | 16F | 1 | 5 | 2 | 2 | 4 | 14 | 6.78 | 0.800 |
| 14 | 16F | 1 | 6 | 2 | 1 | 3 | 13 | 8.38 | 1.040 |
| 15 | 16F | 1 | 1 | 1 | 1 | 2 | 6 | 35.51 | 4.240 |
| 16 | 17M | 2 | 2 | 1 | 1 | 2 | 8 | 20.35 | 2.480 |
| 17 | 19M | 1 | 1 | 1 | 1 | 3 | 7 | 27.93 | 3.360 |
| 18 | 20F | 1 | 11 | 1 | 4 | 4 | 21 | 2.39 | 0.320 |

**Table S4.** **Neck treatment site. Patient demographic, anatomical data and simulation results.**

| Subject # | Age / Gender | Skin, mm | Fat, mm | Muscle, mm | Cartilage, mm | Muscle, mm | Total Tissue Thickness, mm | Fluence Rate, mW/cm2 | Dose @ 1 min, J |
| --- | --- | --- | --- | --- | --- | --- | --- | --- | --- |
|  |  |  |  |  |  |  |  |  |  |
| 1 | 5F | 1 | 2 | 1 | 4 | 1 | 9 | 26.33 | 3.200 |
| 2 | 6M | 1 | 1 | 4 | 4 | 1 | 11 | 17.56 | 2.080 |
| 3 | 6F | 1 | 1 | 2 | 5 | 1 | 10 | 23.54 | 2.800 |
| 4 | 7M | 1 | 2 | 3 | 5 | 1 | 12 | 15.16 | 1.840 |
| 5 | 7M | 1 | 4 | 1 | 8 | 2 | 16 | 9.58 | 1.120 |
| 6 | 7F | 1 | 4 | 5 | 6 | 2 | 18 | 4.39 | 0.560 |
| 7 | 10M | 1 | 1 | 6 | 5 | 2 | 15 | 6.38 | 0.800 |
| 8 | 10F | 1 | 2 | 3 | 3 | 3 | 12 | 11.97 | 1.440 |
| 9 | 11M | 1 | 1 | 5 | 4 | 2 | 13 | 9.98 | 1.200 |
| 10 | 11F | 1 | 8 | 1 | 6 | 1 | 17 | 7.98 | 0.960 |
| 11 | 14M | 1 | 2 | 1 | 9 | 1 | 14 | 14.76 | 1.760 |
| 12 | 15F | 1 | 1 | 1 | 7 | 1 | 11 | 24.74 | 2.960 |
| 13 | 16F | 1 | 2 | 3 | 4 | 3 | 13 | 9.98 | 1.200 |
| 14 | 16F | 1 | 6 | 3 | 5 | 6 | 21 | 2.00 | 0.240 |
| 15 | 16F | 1 | 2 | 2 | 4 | 2 | 11 | 17.16 | 2.080 |
| 16 | 17M | 2 | 2 | 3 | 6 | 2 | 15 | 6.78 | 0.800 |
| 17 | 19M | 1 | 2 | 3 | 6 | 2 | 14 | 9.98 | 1.200 |
| 18 | 20F | 2 | 7 | 2 | 8 | 2 | 21 | 3.19 | 0.400 |

**Simulated Fluence Rates and Absorbed Power**

Fig. S1 – S6 present the fluence rate and absorbed power distribution in subjects with the thinnest and thickest lip, mandible angle, and neck treatment sites, respectively. Graphs depicting fluence rates are color-coded such that higher rates are red, while lower rates are magenta. Absorbed power is color-coded such that higher levels are white, and lower levels are red.


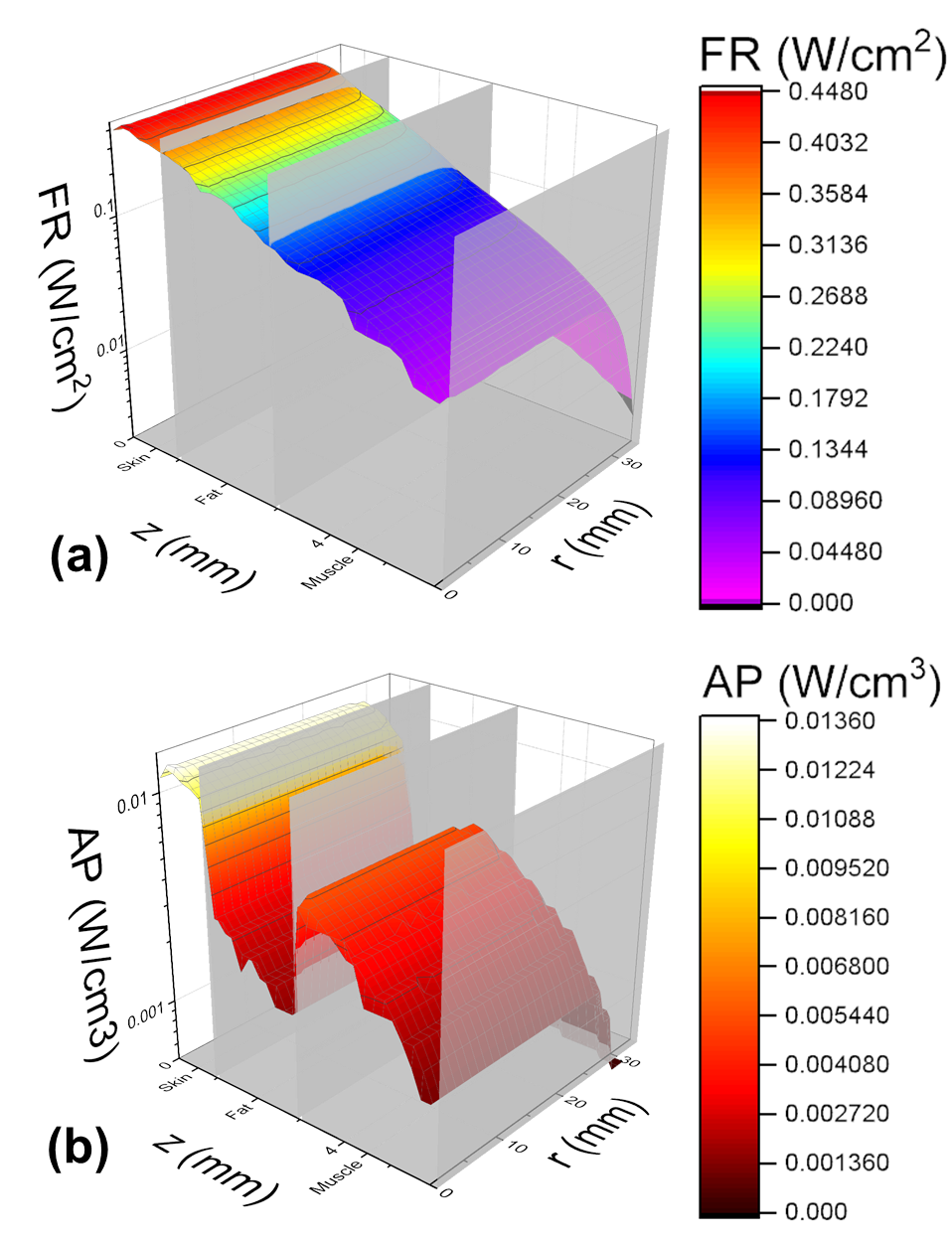


**Fig. S1**. **Fluence Rate and Absorbed Power for Thinnest Lip.** Fluence rate (**a**) and absorbed power (**b**) distributions for Subjects #6 (thinnest lip). The vertical axis corresponds to fluence rate (**a**) or absorbed power (**b**). The z-axis shows treatment volume depth, and the r-axis is the radial distance from the center of the treatment beam.


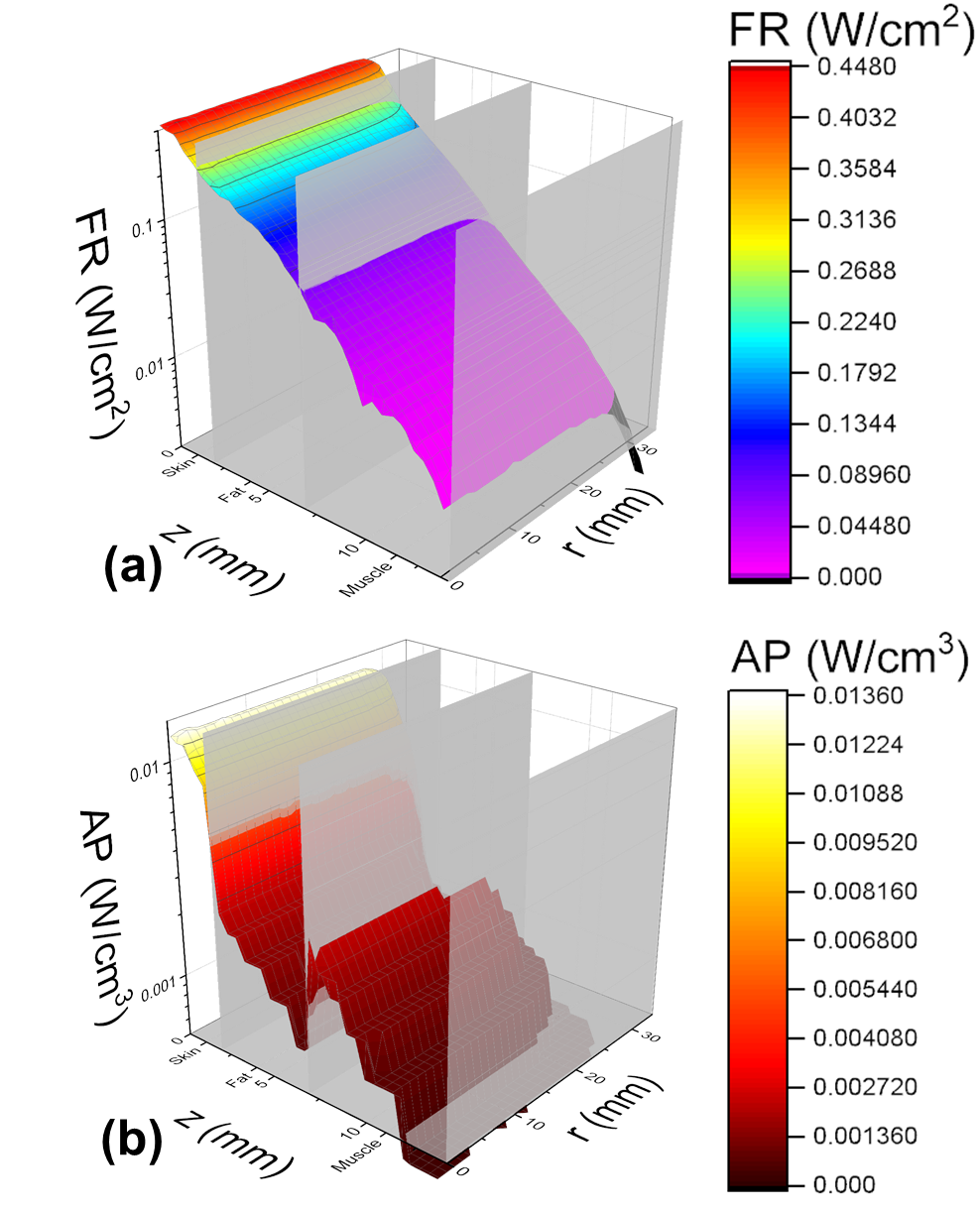


**Fig. S2**. **Fluence Rate and Absorbed Power for Thickest Lip.** Fluence rate (**a**) and absorbed power (**b**) distributions for Subject #16 (thickest lip). The vertical axis corresponds to fluence rate (**a**) or absorbed power (**b**). The z-axis shows treatment volume depth, and the r-axis is the radial distance from the center of the treatment beam.


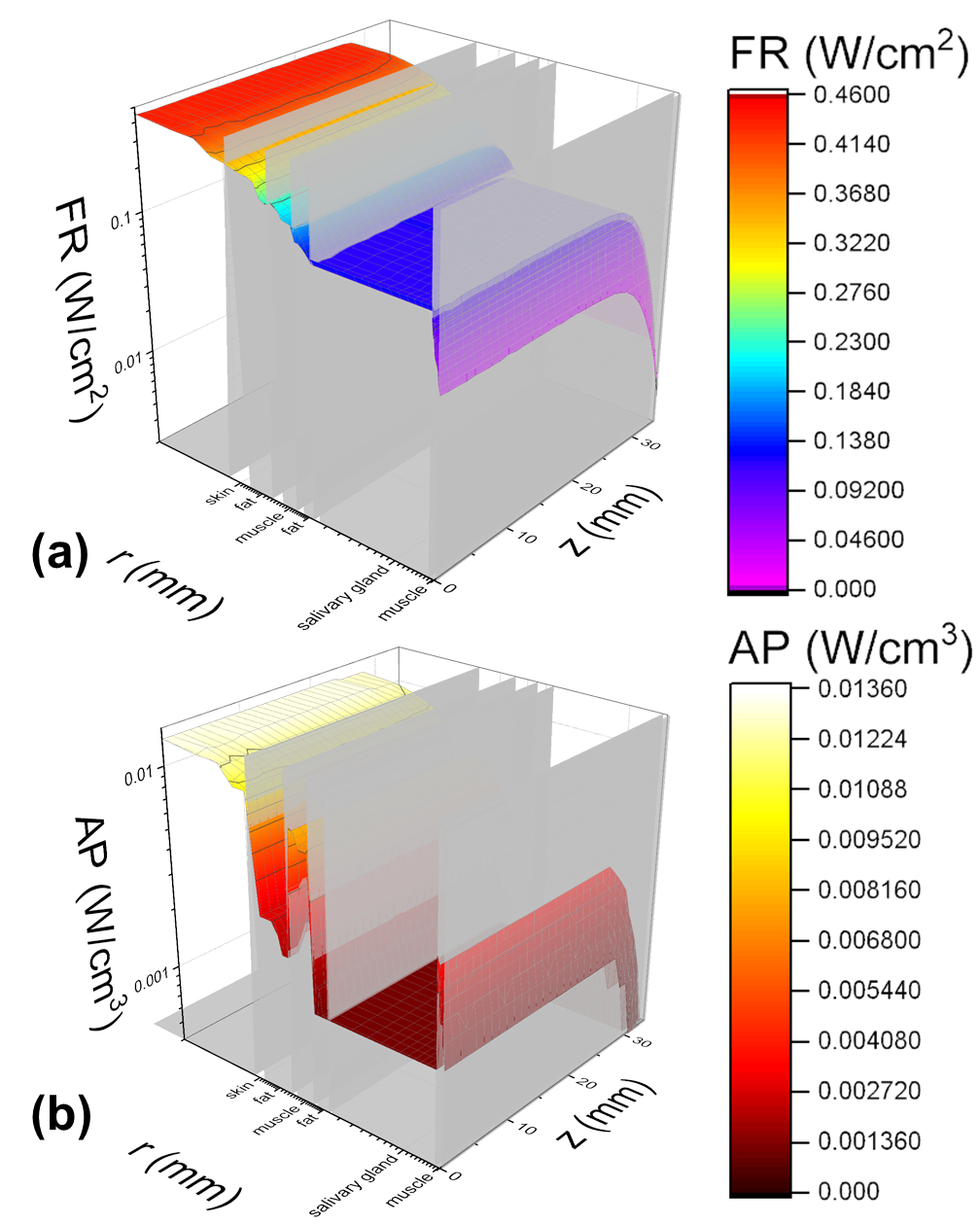


**Fig. S3. Fluence Rate and Absorbed Power for Thinnest Mandible Angle.** Fluence rate (**a**) and absorbed power (**b**) distributions for Subject #15 (thinnest mandible angle site). The vertical axis corresponds to fluence rate (**a**) or absorbed power (**b**). The z-axis shows treatment volume depth, and the r-axis is the radial distance from the center of the treatment beam. Please note that the r-axis is presented in a logarithmic scale.


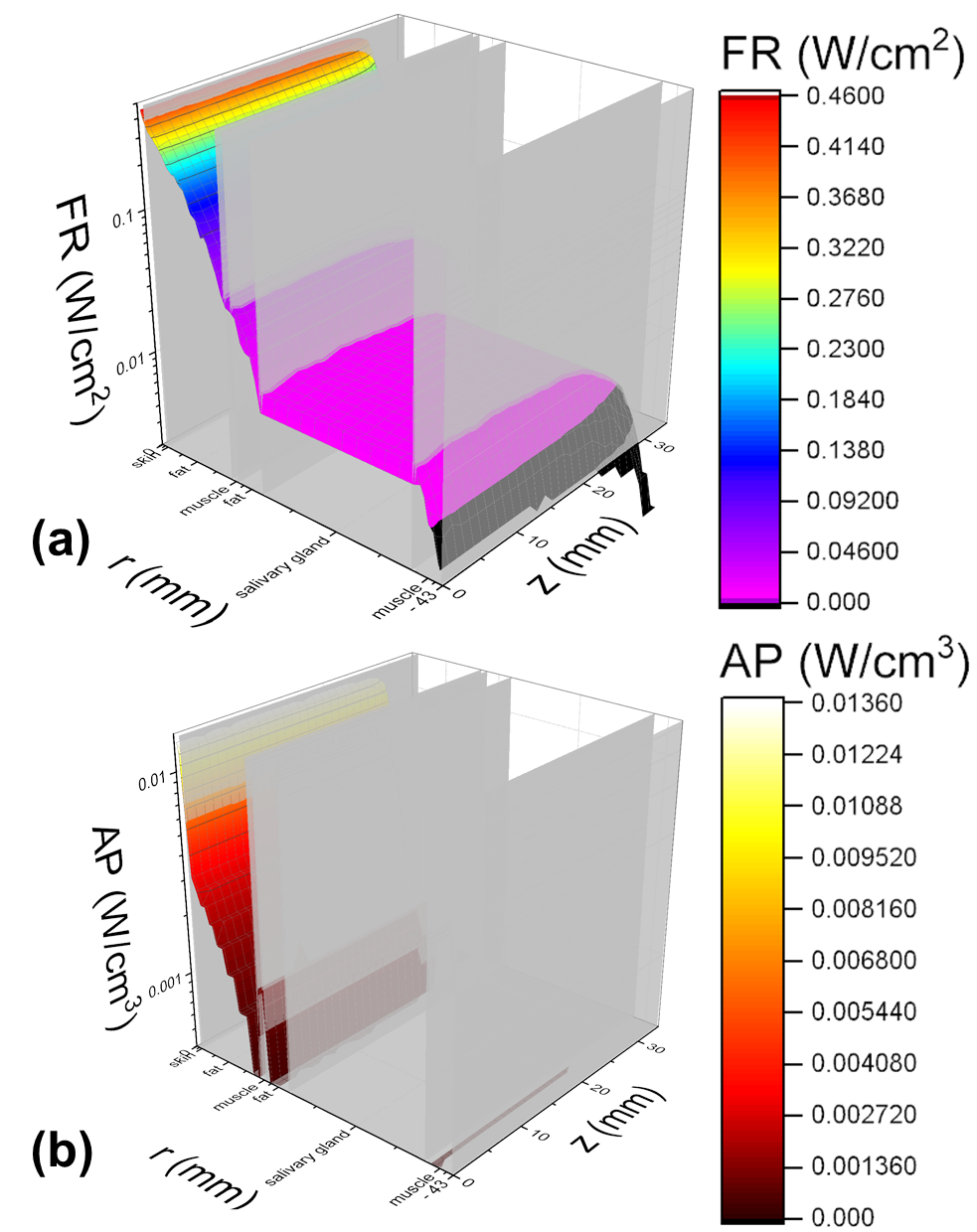


**Fig. S4. Fluence Rate and Absorbed Power for Thickest Mandible Angle.** Fluence rate (**a**) and absorbed power (**b**) distributions for Subject #18 (thickest mandible angle site). The vertical axis corresponds to fluence rate (**a**) or absorbed power (**b**). The z-axis shows treatment volume depth, and the r-axis is the radial distance from the center of the treatment beam.


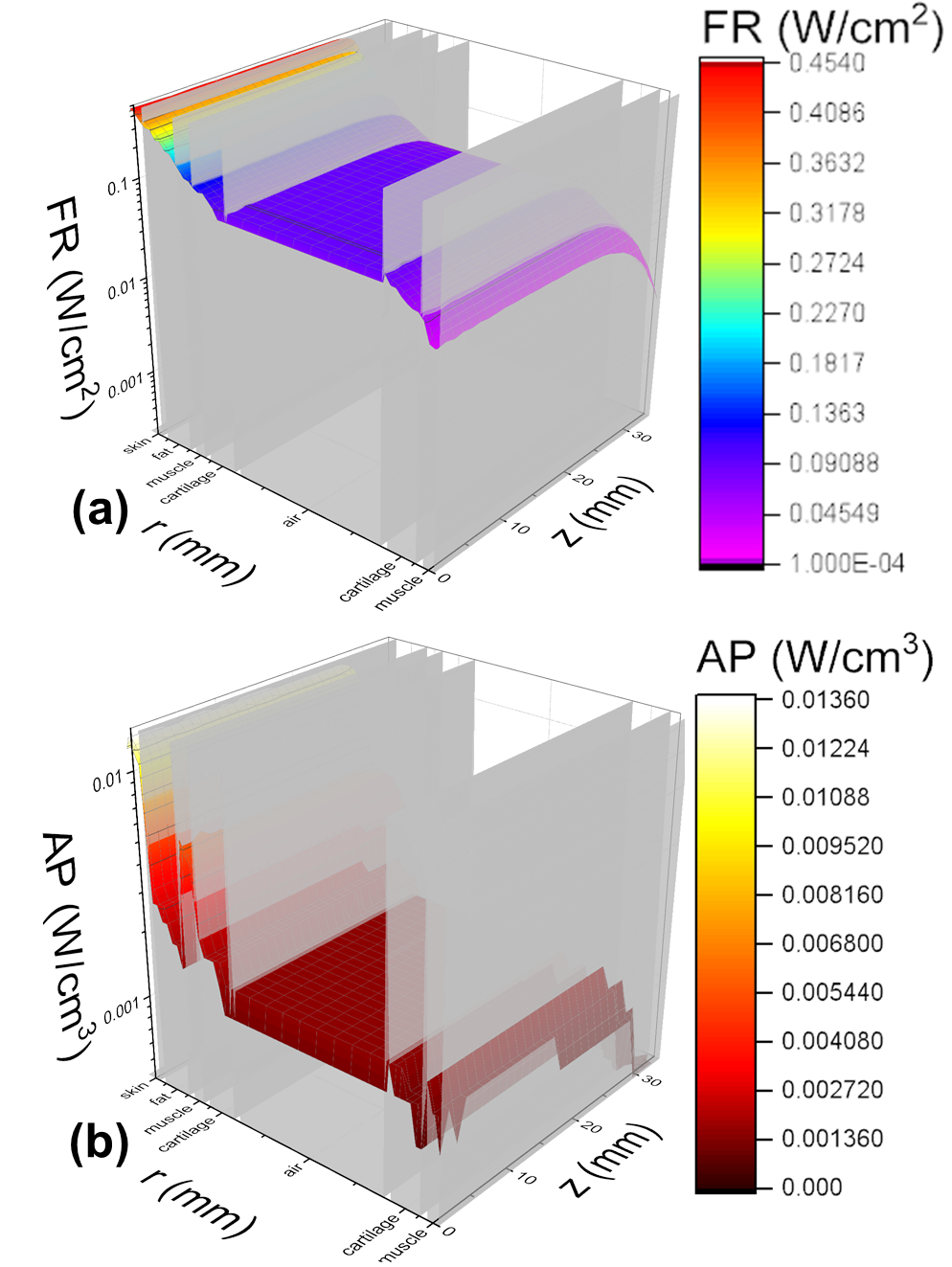


**Fig. S5. Fluence Rate and Absorbed Power for Thinnest Neck.** Fluence rate (**a**) and absorbed power (**b**) distributions for Subject #1 (thinnest neck). The vertical axis corresponds to fluence rate (**a**) or absorbed power (**b**). The z-axis shows treatment volume depth, and the r-axis is the radial distance from the center of the treatment beam.


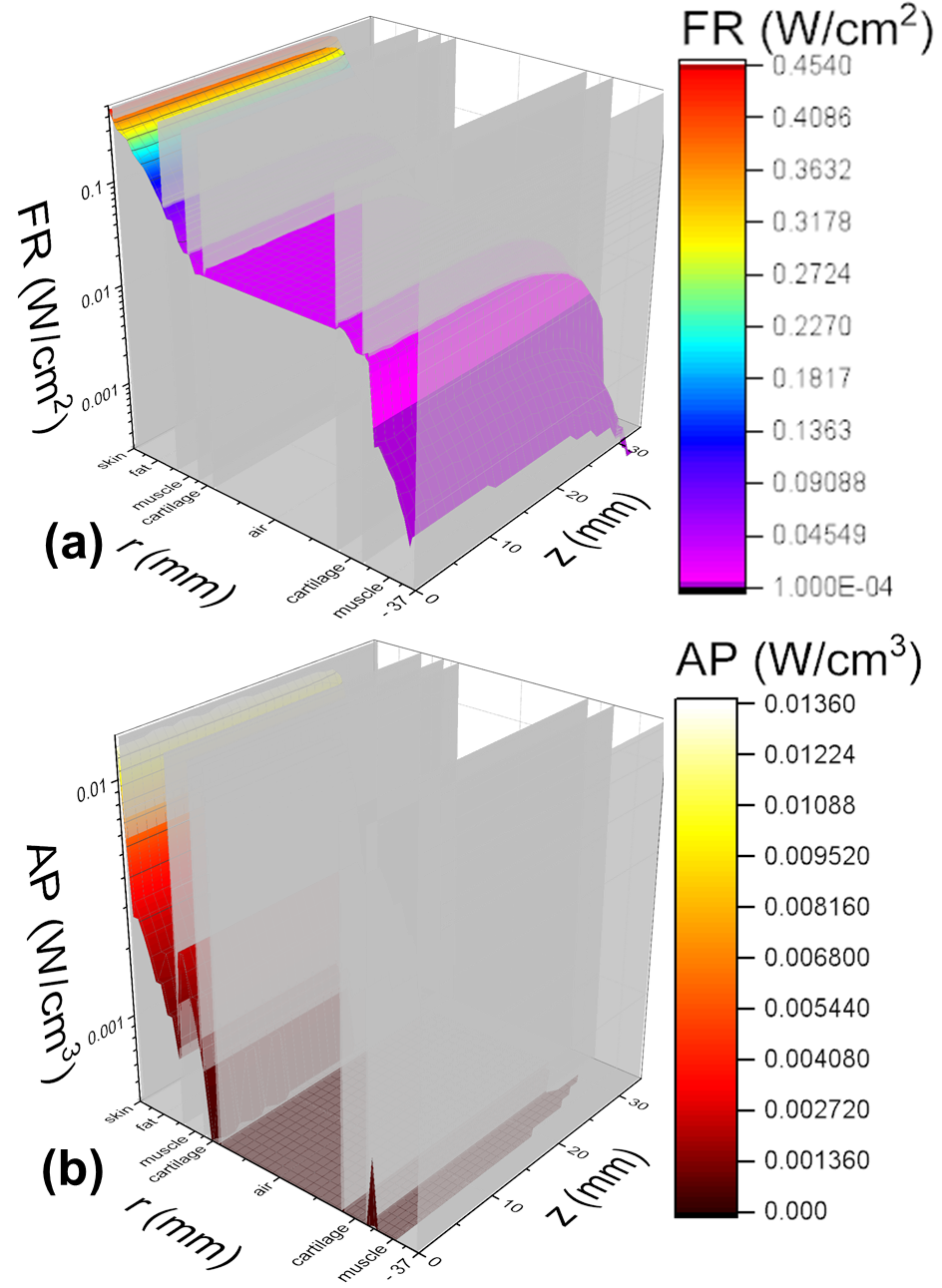


**Fig. S6. Fluence Rate and Absorbed Power for Thickest Neck.** Fluence rate (**a**) and absorbed power (**b**) distributions for Subject #14 (thickest neck). The vertical axis corresponds to fluence rate (**a**) or absorbed power (**b**). The z-axis shows treatment volume depth, and the r-axis is the radial distance from the center of the treatment beam.
